# Construction and verification of prognostic nomogram for early-onset esophageal cancer

**DOI:** 10.17305/bjbms.2021.5533

**Published:** 2021-12

**Authors:** Xiaoxiao Liu, Wei Guo, Xiaobo Shi, Yue Ke, Yuxing Li, Shupei Pan, Yingying Jin, Yuchen Wang, Qinli Ruan, Hongbing Ma

**Affiliations:** Department of Radiation Oncology, Xi’an Jiaotong University Second Affiliated Hospital, Xi’an, China

**Keywords:** Early-onset esophageal cancer (EOEC), nomogram, overall survival (OS), cancer-specific survival (CSS), surveillance, epidemiology, and end results (SEER)

## Abstract

This study aimed to build up nomogram models to evaluate overall survival (OS) and cancer-specific survival (CSS) in early-onset esophageal cancer (EOEC). Patients diagnosed with esophageal cancer (EC) from 2004 to 2015 were extracted from the Surveillance Epidemiology and End Results (SEER) database. Clinicopathological characteristics of younger versus older patients were compared, and survival analysis was performed in both groups. Independent related factors influencing the prognosis of EOEC were identified by univariate and multivariate Cox analysis, which were incorporated to construct a nomogram. The predictive capability of the nomogram was estimated by the concordance index (C-index), calibration plot, receiver operating characteristic (ROC) curve, and decision curve analysis (DCA). A total of 534 younger and 17,243 older patients were available from the SEER database. Younger patients were randomly segmented into a training set (n = 266) and a validation set (n = 268). In terms of the training set, the C-index of the OS nomogram was 0.740 (95% CI: 0.707-0.773), and that of the CSS nomogram was 0.752 (95% CI: 0.719-0.785). In view of the validation set, the C-index of OS and CSS were 0.706 (95% CI: 0.671-0.741) and 0.723 (95% CI: 0.690-0.756), respectively. Calibration curves demonstrated the consistent degree of fit between actual and predicted values in nomogram models. From the perspective of DCA, the nomogram models were more beneficial than the tumor-node-metastasis (TNM) and the SEER stage for EOEC. In brief, the nomogram models can be considered as an individualized quantitative tool to predict the prognosis of EOEC patients to assist clinicians in making treatment decisions.

## INTRODUCTION

Esophageal cancer (EC) is one of the most aggressive gastrointestinal tumors [1]. EC is the seventh leading cancer type for males in the United States. According to cancer statistics in 2020 manifested that there were approximately 18,440 new cases of EC and 16,170 death from EC, while in 2019 there were about 17,650 new cases of EC [2,3]. It is evaluated that the global incidence and mortality rate of the EC will increase in the future years, particularly in Asia [4]. What’s more, the morbidity of esophageal adenocarcinoma has increased significantly in all ages, especially among young people [5]. Besides, some studies have reported that there was no distinction in the survival rate between young and elderly patients with EC, but young patients with EC have more advanced tumors and more malignant potential and invasiveness than older patients [6]. A relevant study had manifested that compared with elderly patients, while patients with early-onset esophageal adenocarcinoma (younger than 50 years) were a high proportion of advanced stages; they had a superior survival rate [7]. Furthermore, due to being often diagnosed at an advanced stage at the time of consultation, esophageal cancer has a poor prognosis [8]. It is still controversial whether younger patients with EC have a better or worse prognosis than older patients, which draws our attention to which clinicopathological factors affect the prognosis of young patients with esophageal cancer [9-15].

According to the age group determined by the World Health Organization (WHO) in 2017, the upper age limit for young people has been raised to 44 years old. For the time being, there is no acknowledged explicit definition of early-onset EC. Therefore, in this study, early-onset esophageal cancer (EOEC) referred to patients with age ≤44 years old.

The TNM staging system is considered to be currently the most extensively used system for prognostic evaluation and clinical treatment of cancer patients. It contains tumor invasive depth, regional lymph node involvement, and distant metastasis, but does not contain demographic information about patients, such as age, gender, race, food habit, and marital status, which are also concerned with the prognosis of cancer patients, resulting in that the TNM staging system cannot thoroughly predict the prognosis of EC patients [16]. Consequently, the main purpose of this study was to develop more plentiful and accurate prognostic models to guide survival. Nomogram can evaluate and analyze the risk factors of prognosis visually and individually [16-18]. However, the nomogram of the prognosis of EOEC has not been completely determined.

In this research, we made use of data from the SEER (Surveillance, Epidemiology, and End Results) database to first probe into the differences in clinicopathological characteristics influencing the prognosis of younger and older patients. We then focused on analyzing the clinicopathological features of EOEC and thoroughly investigating the variables associated with prognosis. Ultimately, we further constructed nomogram models to preferably forecast the prognosis of EOEC.

## MATERIALS AND METHODS

### Patients

The National Cancer Institute’s SEER database (http://seer.cancer.gov/seerstat/) covers 28% of the population of the United States. This study used SEER database data from 1975 to 2016 (“Incidence - SEER 18 Regs Custom Data (with additional treatment fields), Nov 2018 Sub (1975-2016 varying)”). Data were extracted, downloaded, and analyzed using SEER*Stat Software (Version 8.3.6).

Patients diagnosed with EC from 2004 to 2015 were retrieved. Inclusion criteria included the following: (I) EC patients proved by pathology; (II) only one primary tumor; and (III) complete clinicopathological data and follow-up information. Exclusion criteria were as follows: (I) The patient’s disease-related information is missing, such as unknown age, sex, race, grade, primary site, TNM stage, SEER stage, and other information; and (II) unknown surgery, radiotherapy, and chemotherapy.

Patients were staged using the American Joint Committee on Cancer (AJCC) version 6^th^. In this study, the upper esophagus was set up with primary site codes C15.0 (cervical esophagus) and C15.3 (upper third of esophagus). Code C15.1 ­(thoracic esophagus) and C15.4 (middle third of esophagus) were considered to be the middle esophagus. The lower esophagus was defined with codes C15.2 (abdominal esophagus) and C15.5 (lower third of esophagus) [19].

**Figure 1:**
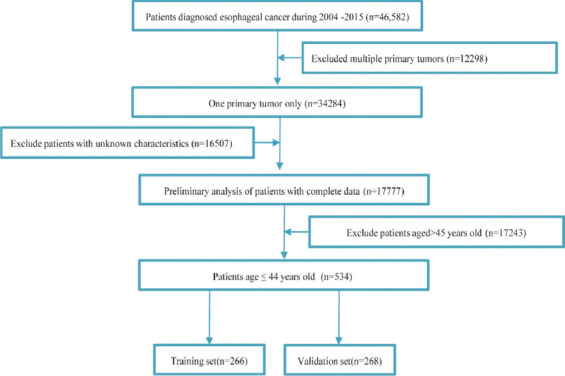
Flow diagram of EOEC patient screening.

In the end, a total of 17777 patients with EC met the inclusion and exclusion criteria, which included 17,243 elderly patients older than 45 years and 534 patients with EOEC. For further analysis, the selected patients with EOEC were divided into the training set (n = 266) and a validation set (n = 268) at random (Figure 1).

### Variables

Clinical variables captured from the SEER database encompassed age, sex, race, grade, histology, primary site, TNM 6^th^ stage, SEER stage, surgery, radiotherapy, and chemotherapy. Follow-up variables involved survival status, survival time, and cause of death. The principal terminal point was overall survival (OS), which referred to the period from diagnosis to death for any reason. Nevertheless, cancer-specific survival (CSS) was the subordinate terminal point, which was regarded as the period from diagnosis to death owing to the EC. All patients were followed up for at least 3 months.

### Construction and verification of nomogram

Significant variables screened by univariate analysis were further screened out independent prognostic factors by multivariate analysis and then the nomogram was constructed. The concordance index (C-index), receiver operating characteristics (ROC) curves, and calibration curves were used to evaluate the performance and accuracy of nomogram [20]. Moreover, the nomogram was compared with the TNM stage and SEER stage utilizing decision curve analysis (DCA) that was a novel way for evaluating predictive models [21,22].

### Ethical statement

Data in the present study could be freely obtained from the SEER database, which was utilized and analyzed by the public (http://seer.cancer.gov/seerstat/). Therefore, the study exempted the institutional review board approval.

### Statistical analysis

Chi-square test was used for comparison of categorical variables, and the comparison of ordinal variables was based on the Wilcoxon rank-sum test. The Kaplan–Meier method was utilized for demonstrating the effect of clinicopathological variables on the survival rate of patients. The inspection level was defined as *p* < 0.05, which represented that the difference was considered dramatically significant. All statistical analyses and drawings were implemented utilizing R software version 3.6.2 (http://www.R-project.org).

## RESULTS

### Comparison of clinical characteristics between younger and older of EC

**Figure 2:**
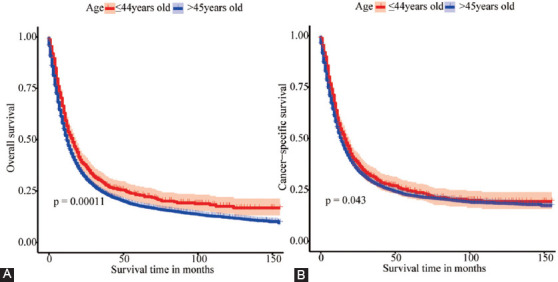
Kaplan-Meier survival curves of different age groups of esophageal cancer. (A) The survival curve for overall survival (OS) was compared between patients ≤44 and >45 years of age. (B) The survival curve for cancer-specific survival (CSS) was compared between patients ≤ 44 and >45 years of age.

As indicated in Table 1, we enrolled a total of 534 younger and 17,243 older patients with EC after screening. Older patients accounted for the majority of the entire cohort. Nevertheless, the number of younger patients represented only 3.1% of the older patients. Statistical analysis revealed significant differences in, race (*p* = 0.043), histology (*p* < 0.001), primary site (*p* < 0.001), T (*p* < 0.001), N (*p* < 0.001), M (*p* < 0.001), SEER stage (*p* < 0.001), surgery (*p* < 0.001), and chemotherapy (*p* < 0.001) between patients less than or equal to 44 years of age and older than 45 years. Regardless of age, male patients were more likely than female patients. However, there were more white people in the younger group (81.46%), while the older group was more black people (84.99%). The depth of tumor immersion was mainly T3, which accounted for 41.39% and 40.06%, respectively, in the younger and older groups. Moreover, it was shown that patients with EC, young or not, were more inclined to receive adjuvant radiotherapy and chemotherapy. However, radiotherapy was not statistically significant in both age groups of patients (*p* = 0.859), which might be due to the lack of detailed radiotherapy information in the SEER database. Survival analysis illustrated that the medium survival time of young and old patients was 15.00 (6.00-33.00) and 12.00 (5.00-27.00) months, respectively (*p* < 0.001). The survival curves suggested that the OS and CSS of younger patients were higher than those of older patients, and the differences were statistically significant (*p* < 0.001, *p* = 0.043) (Figure 2). Although our study found that the younger group had a better prognosis than the older group, the study of EC patients aged ≤44 years has aroused widespread concern with the speedy ascent in the incidence. Therefore, we next investigated the prognostic evaluation models in the younger patients.

**TABLE 1:**
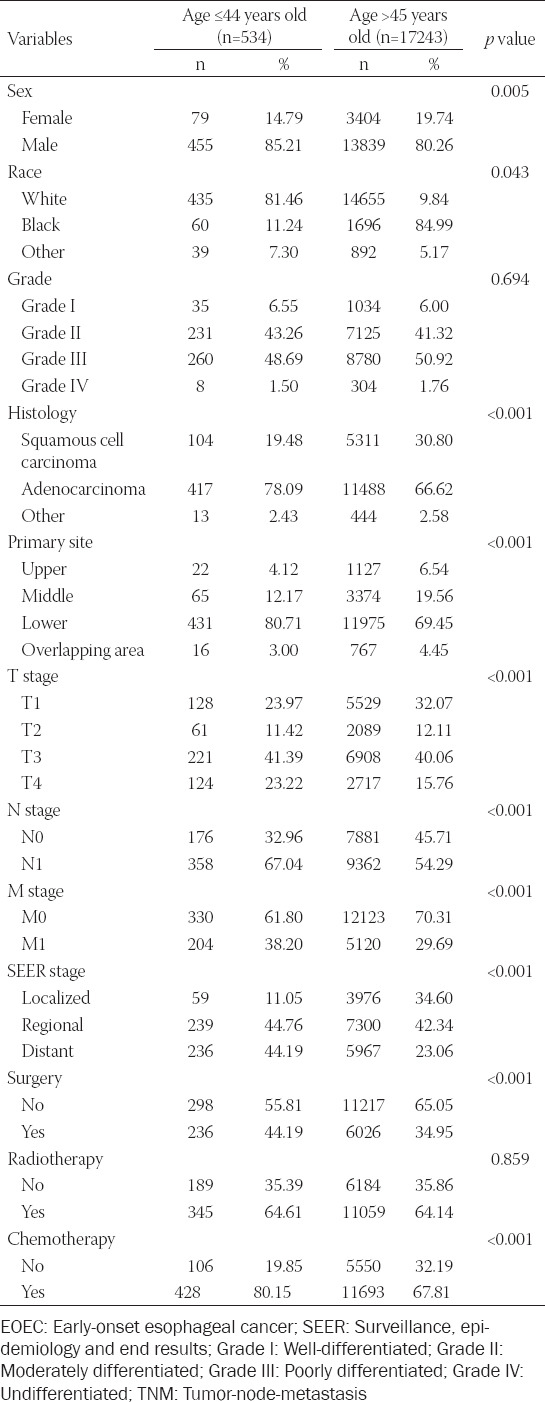
Comparison of demographic and clinicopathological characteristics of EC in younger and older patients

### Confirmation of the cut-off age

**Figure 3:**
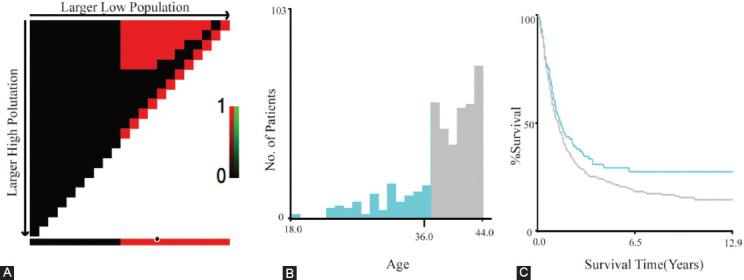
The X-tile analysis was implemented on the survival data of EOEC patients to ascertain the optimal threshold for age. The best cutting point was highlighted with a black circle in the left figure 3A. Histogram of the entire group in middle Figure 3B, and Kaplan-Meier plot (C). The figure manifested that the optimal age cut-off point for EOEC patients was 37 years (≤ 36, 37 – 44, p < 0.001).

X-tile software analysis result displayed that the optimal cut-off age in EOEC patients was 37 years; therefore, the patients were fell into two groups (≤36, 37-44, Figure 3) [23].

### Characteristics of patients with EOEC

**Figure 4:**
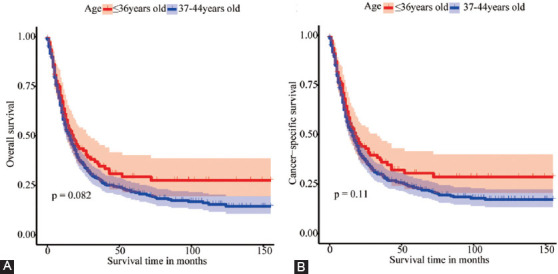
Kaplan-Meier survival curves of different age groups of EOEC. (A) The survival curve for OS was compared between patients≤36 and 37-44 years of age. (B) The survival curve for CSS was compared between patients≤36 and 37-44 years of age.

Overall, 534 EOEC patients qualified were involved in this study, of which 266 patients were randomly allocated to the training set and 268 patients into the validation set (Figure 1). Notably, there was no significant difference between the two sets. Amid all EOGC patients, 425 (79.59%) were between 37 and 44 years of age, while 109 (20.41%) were under 37 years of age. Similar to previous reports, males (85.21%) accounted for significantly more than females (14.79%). The race was predominantly white (81.46%). Grades were dominated by Grade II (43.26%) and Grade III (48.69%). The most common histology type was adenocarcinoma (78.09%). Moreover, the most frequent tumor location of EOGC was the lower esophagus (80.71%), afterward the middle esophagus (12.17%) and the upper esophagus (4.12%). For all the patients, T3 and N0 account for 41.39% and 32.96%, respectively, while 61.80% is M0 in the AJCC stage system. Concerning the SEER stage, there were 239 patients (44.76%) of regional and 236 patients of distant that accounted for 44.19% (Tables 1 and S1). Our analysis resulted in medium survival time of 16.00 (7.00-40.00) and 14.00 (6.00-31.00) months for patients ≤36 years and 37-44 years, respectively (p = 0.304). Kaplan–Meier survival curves demonstrated that patients aged 37-44 years had lower OS and CSS than patients aged ≤36 years. However, the differences were not statistically significant (*p* = 0.082, *p* = 0.11) (Figure 4).

### Exploration of prognostic factors associated with OS and CSS

Univariate analysis demonstrated that age, race, grade, histology, primary site, TNM stage, SEER stage, surgery, and radiotherapy were significantly correlated with OS (Table 2). Whereas, among the multivariate analysis, age, grade, histology, primary site, SEER stage, and surgery were regarded as independent hazard factors for OS. The result of CSS univariate analysis manifested that age, race, grade, histology, primary site, TNM stage, SEER stage, surgery, and radiotherapy were prognostic risk factors for EOEC patients in the training set (Table 3). The results of our analysis also manifested that although the *p*-value of the primary site was 0.05, as we know, it also affected the prognosis of patients, so we also included this factor of the primary site in the multivariate analysis. In multivariate analysis, age, race, grade, histology, primary site, SEER stage, and surgery were significantly correlated with CSS.

**TABLE 2:**
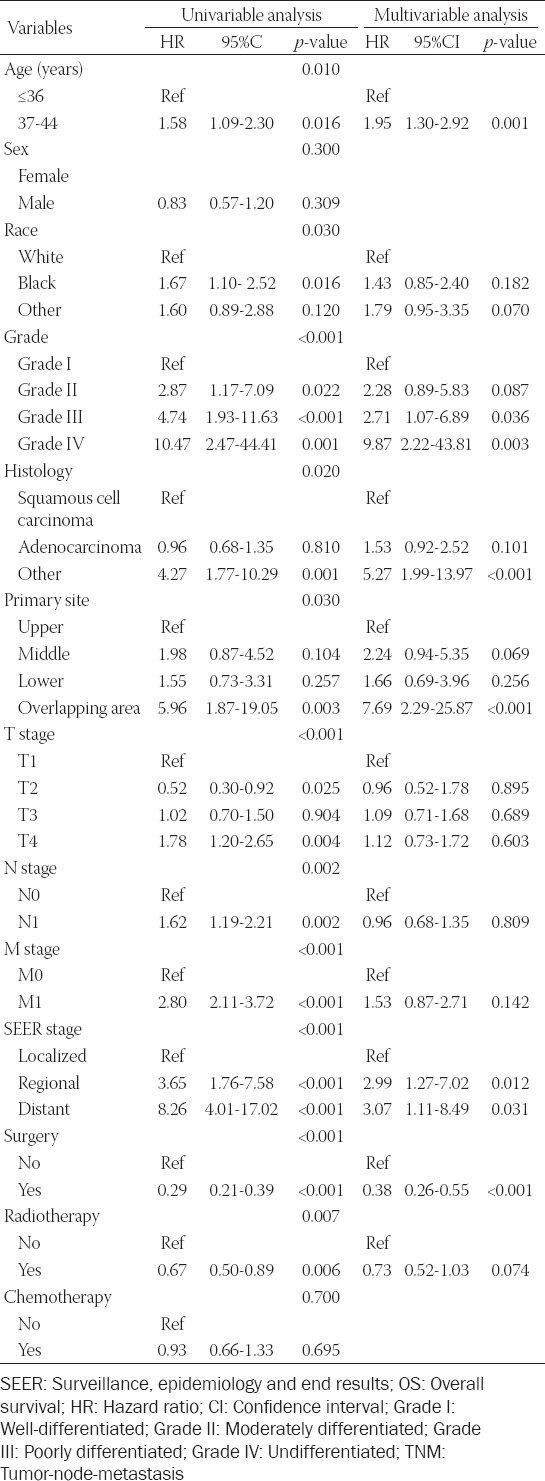
Univariate and multivariate analysis of variables related to OS in the training set (n=266)

**TABLE 3:**
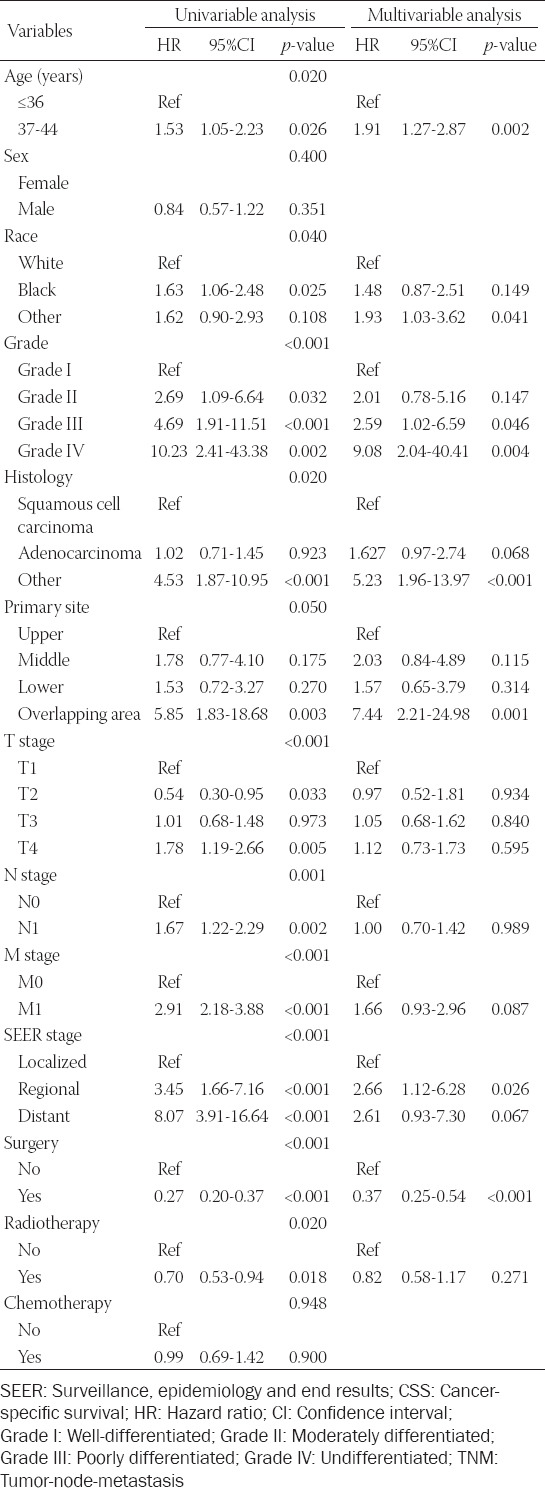
Univariate and multivariate analysis of variables related to CSS in the training set (n=266)

### Construction of the nomogram

**Figure 5:**
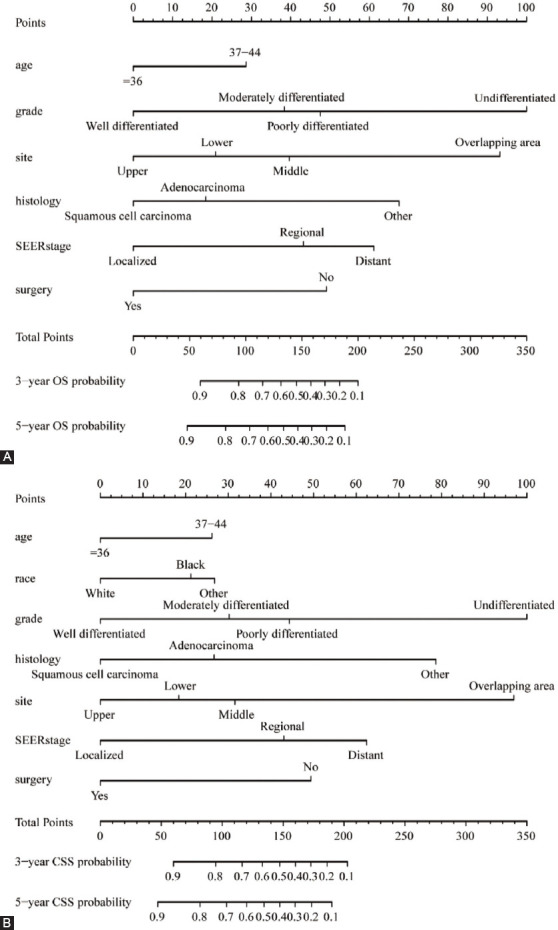
Nomogram models of OS and CSS for EOEC patients in the training set. (A) Nomogram models of 3-year and 5-year OS for patients with EOEC. (B) Nomogram models of 3- and 5-year CSS for EOEC patients.

The nomogram of OS was constructed by incorporating prognostic risk factors that contained age, grade, histology, primary site, SEER stage, and surgery given by performing the multivariate analysis (Figure 5A). Simultaneously, variables were significantly correlated with CSS, which was further included in the CSS nomogram (Figure 5B). The scores corresponding to independent risk factors were summed to calculate the aggregate score. According to the total score value, the predicted probabilities of OS and CSS in 3 and 5 years could be obtained.

### Verification of the nomogram

**Figure 6:**
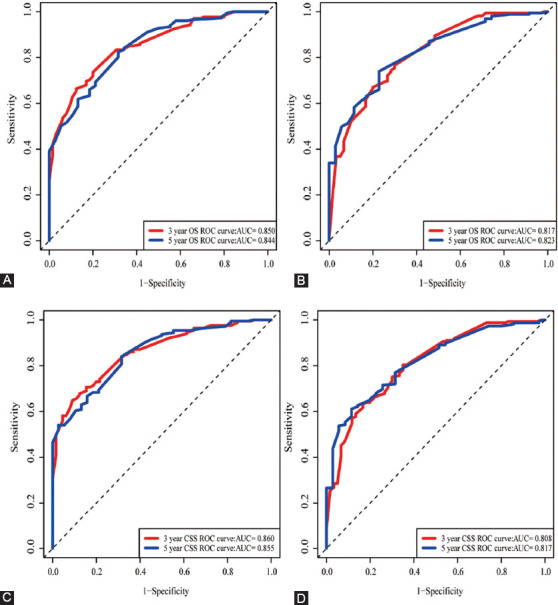
The area under the receiver operating characteristic (ROC) curve (AUC) was utilized to weigh up the performance of OS and CSS nomogram models. (A, B) ROC curves for 3- and 5-year OS in the training set and validation set; (C, D) ROC curves for 3- and 5-year CSS in the training set and validation set.

**Figure 7:**
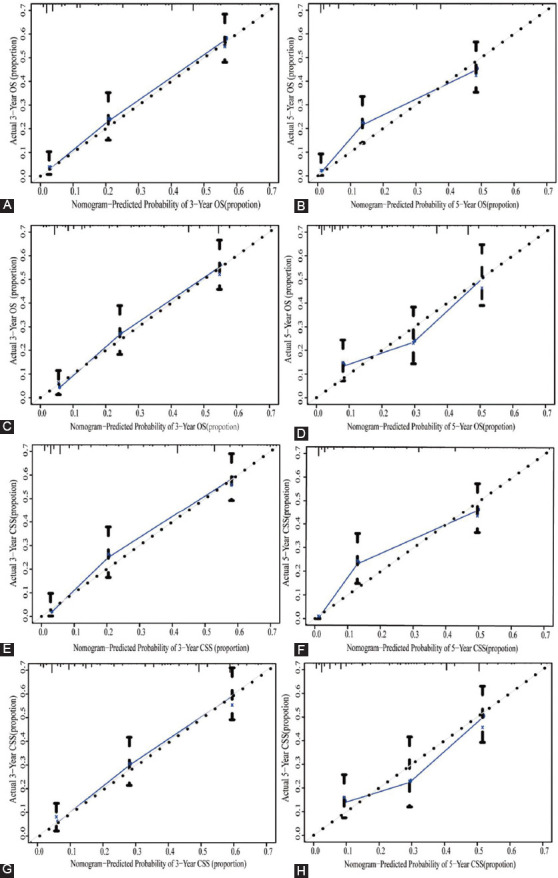
Calibration curves for nomogram models related to OS and CSS. (A, B) Calibration curves for 3- and 5-year OS in the training set; (C, D) Calibration curves for 3- and 5-year OS in validation set; (E, F) Calibration curves for 3- and 5-year CSS in the training set; (G, H) Calibration curves for 3- and 5-year CSS in the validation set.

The OS and CSS nomogram models were verified internally through the training set and externally by the validation set, respectively. According to internal verification, the C-index of OS nomogram was 0.740 (95% CI: 0.707-0.773), and that of CSS nomogram was 0.752 (95% CI: 0.719-0.785). In terms of the external verification, the C-index of OS and CSS were 0.706 (95% CI: 0.671-0.741) and 0.723 (95% CI: 0.690-0.756), respectively. The area under the ROC curves (area under curve, AUC) that were applied to evaluate the discernment of the nomogram models were both high in the training set and verification set (Figure 6). Additionally, the calibration curves were relatively close to the ideal curve, which indicated the probabilities of 3-year and 5-year OS and CSS forecasted by nomogram models were in accordance with the actual survival of patients (Figure 7).

**Figure 8:**
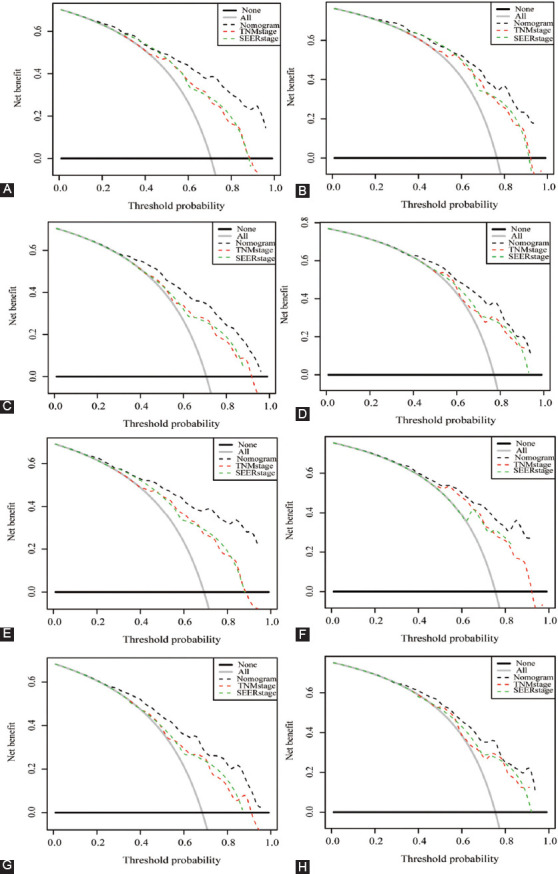
Decision curve analysis (DCA) for nomogram, TNM 6^th^ stage, and SEER stage. (A, B) DCA for 3- and 5-year OS in the training set; (C, D) DCA for 3- and 5-year OS in validation set; (E, F) DCA for 3- and 5-year CSS in the training set; (G, H) DCA for 3- and 5-year CSS in the validation set.

What is more, we also performed a contrast of the nomogram with the AJCC TNM stage and SEER stage. First of all, the C-index of OS nomogram in training set was 0.740, which was obviously superior to the TNM 6^th^ stage (0.692, 95% CI: 0.653- 0.731; *p* = 0.006) and SEER stage (0.667, 95% CI: 0.631-0.702; *P* < 0.001). Likewise, the C-index of the CSS nomogram in the training set was 0.752, which was significantly better than the TNM 6^th^ stage (0.689, 95%CI: 0.652-0.726; *p* < 0.001) and SEER stage (0.667, 95% CI: 0.632-0.702; *p* < 0.001). Compared with TNM 6^th^ stage and SEER stage, the DCA results of nomogram models had advanced net benefits, which demonstrated that nomogram models had superior clinical prognostic worth than TNM 6^th^ stage and SEER stage (Figure 8).

## DISCUSSION

In this research, we extracted a total of 17777 EC patients with complete information from the SEER database, of which contained 17,243 elderly patients older than 45 years and 534 patients with EOEC. Subsequently, we explored the differences in clinicopathological factors affecting the prognosis of younger and older patients and carried out survival analysis, which found that the survival rate of younger was higher than that of older patients. As incidence increased in young people worldwide, we consequently focused on investigating prognostic risk factors for EOEC in this study. We randomly divided 534 patients with EOEC into two groups, of which 266 patients were regarded as the training group and 268 patients were considered to be the external validation group. The independent hazard factors relevant to OS and CSS were identified by univariate and multivariate analysis. We further integrated these factors into the nomogram to forecast probabilities of OS and CSS in 3 and 5 years, which demonstrated high accuracy of this nomogram through internal and external validation. Compared with the TNM stage and SEER stage, the nomogram was more predictive, guiding the prognosis evaluation of EOEC patients in terms of visualization and individualization.

EC is one of the most mortality cancers worldwide [3]. According to the Global Burden of Disease (GBD) analysis, the topmost burden of EC (from the standpoint of disability-adjusted life-years) was in East Asia, notably China [24]. In addition, the relevant GBD study found that the age-standardized mortality and incidence rates of EC in China were 2.1 times higher than the global average [25]. A relevant study had reported that risk factors for EC included poor dietary habits such as smoking, alcohol consumption, low fruit intake, and eating spicy foods [26]. As with previous reports, our research also found that EOEC was predominantly male and adenocarcinoma was the dominant histological type of EOEC. Relevant study reported androgen receptor regulated the growth of esophageal adenocarcinoma in a paracrine manner [27]. The incidence of adenocarcinoma of EC in developed countries in Europe and the United States had exceeded that of squamous cell carcinoma that was the predominant histological subtype of esophageal cancer in Central Asia [24,28,29]. Moreover, our findings manifested that the primary site of EOEC was mainly concentrated in the abdominal esophagus and the lower third of the esophagus, which might be adjacent to the stomach where adenocarcinoma was prone to occur. Moreover, it was worth noting that gender was not an independent risk factor for EOEC patients, which was consistent with the findings of Zeng et al. [30]. Multivariate analysis indicated that age, grade, histology, primary site, SEER stage, and surgery were significantly relevant to OS, which was considered as independent predictors for EOEC. In addition, the independent predictors of CSS for EOEC also included race and radiotherapy.

As shown in the nomogram models, the grade had the greatest impact on prognosis in this study. The more severe the differentiation of EOEC, the worse the prognosis. As the SEER stage increased, the tumor gradually progressed, the survival time gradually decreased, and the prognosis became worse, which was the same trend obtained from the nomogram models. It had attracted our attention that nomogram demonstrated that the primary site was also a weighty variable in the prognosis of EOEC patients, especially lower esophageal that was prone to Barrett’s esophagus, which was closely related to gastroesophageal reflux disease (GERD) that was regarded as a precancerous lesion [31]. It is well-known that the main treatment of cancer was surgery, chemotherapy, and radiotherapy. Endoscopic resection (ER) was used to treat the early-stage of EC, while chemoradiotherapy was commonly used for locally advanced EC [32]. Our results also validated the importance of surgery, which improved the prognosis of patients with EOEC from the nomogram. It is generally considered that different chemoradiotherapy regimens have different efficacy and may also have different outcomes. What is more, radiotherapy and chemotherapy variables were not included in our prognostic nomogram models. It might be due to the lack of detailed radiotherapy and chemotherapy protocol information in the SEER database. In recent years, targeted therapy has become increasingly hot, and it refers to novel treatments developed by blocking immune checkpoints. Clinical trials of programmed death-1 (PD-1) and its ligand (PD-L1) inhibitors in the treatment of EC had manifested that compared with monotherapy, the combination therapy improved the survival rate, and significantly benefit patients [33,34].

The limitations of this study were several aspects. First and foremost, the SEER database provided limited information on chemoradiotherapy specific treatment regimens, targeted therapies, and genomic status, which affected prognosis. The genes with the highest frequency of mutations in early-onset esophageal adenocarcinoma were TP53 (73%) and P16 (16%), and other mutations occurred only in: APC, CDH1, CTNNB1 FGFR2, and STK11 [35]. Obesity was concerned with the early-onset of gastrointestinal cancer [36]. Furthermore, the data of this study were all from the United States, and the study would be more meaningful if data from China were further verified. What is more, our findings might be influenced by patients’ willingness to treat. Most important of all, this study was a retrospective study based on the SEER database and required further verification with a prospective cohort study to have sufficient evidence to verify the findings.

Nevertheless, the C-index, AUC, and calibration curves were usually utilized to weigh up the nomogram, which proved that the nomogram had advanced accuracy. Compared with the traditional staging, DCA illuminated that the nomogram had better practicability.

## CONCLUSION

In conclusion, the nomogram models were available for an individualized quantitative implement to predict the prognosis of EOEC patients to assist clinicians in making treatment decisions.

## Figures and Tables

**TABLE S1:**
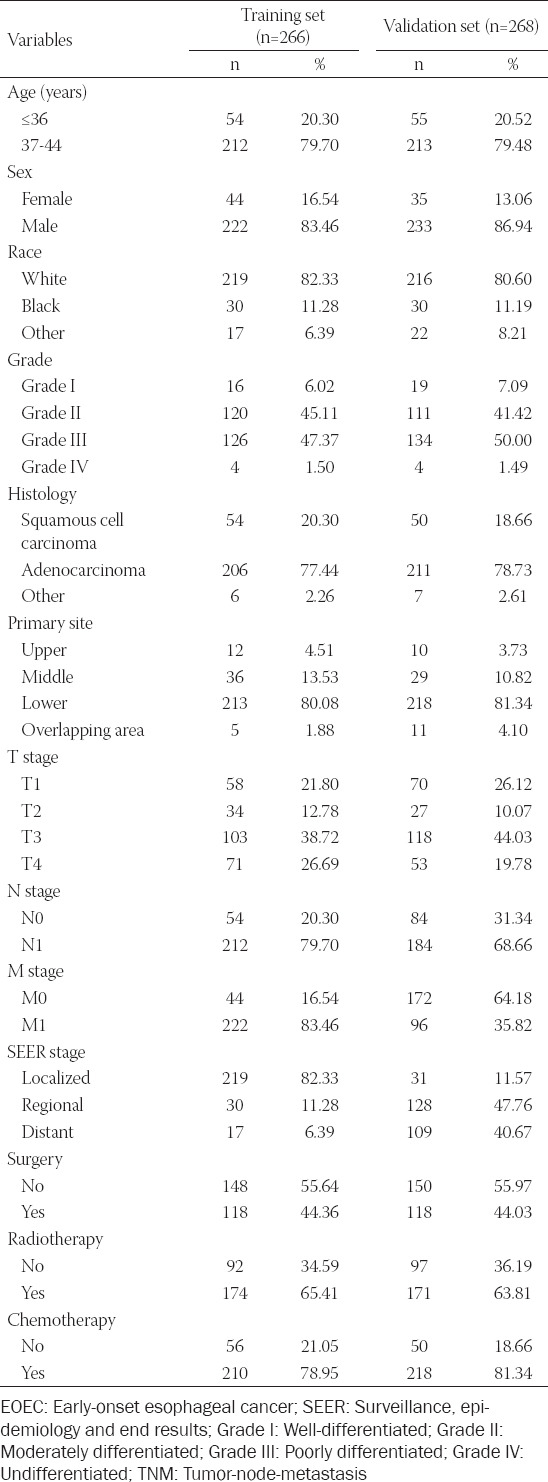
Demographic and clinicopathologic characteristics of EOEC patients
